# Antimicrobial stewardship effectiveness on rationalizing the use of last line of antibiotics in a short period with limited human resources: a single centre cohort study

**DOI:** 10.1186/s13104-019-4572-x

**Published:** 2019-08-20

**Authors:** Darija Kuruc Poje, Vesna Mađarić, Vlatka Janeš Poje, Domagoj Kifer, Philip Howard, Srećko Marušić

**Affiliations:** 1 Hospital Pharmacy Ward, General Hospital “Dr. Tomislav Bardek”, Željka Selingera 1, 48 000 Koprivnica, Croatia; 2 Pulmology and Infectology Ward, General Hospital “Dr. Tomislav Bardek”, Željka Selingera 1, 48 000 Koprivnica, Croatia; 3 Clinical Microbiology Ward, Department of Public Health Koprivnica County, Željka Selingera 1, 48 000 Koprivnica, Croatia; 4 Department of Biophysics, Faculty of Pharmacy and Biochemistry, University of Zagreb, A Kovačića 1, 10 000 Zagreb, Croatia; 5 Leeds Institute of Diagnostics and Therapeutics, Faculty of Medicine and Health, University of Leeds, Leeds, LS2 9JT UK; 6 Department of Endocrinology, Diabetes and Metabolic Diseases, Clinical Hospital Dubrava, Avenija Gojka Šuška 6, 10 000 Zagreb, Croatia

**Keywords:** Antibiotic reserve, Antimicrobial stewardship, Multidisciplinary approach, Limited human resources, Croatia

## Abstract

**Objective:**

Antibiotics reserve (ARs) are given as a last line of treatment when other antibiotics are no longer effective. Rising threat of antimicrobial resistance makes growing use of ARs a real problem to patient safety. A single centre interventional cohort study was conducted in order to measure impact on clinical outcomes of A-team programme with limited human resources in a short period. A-team programme started on 01. September 2017.

**Results:**

In 3 months preintervention and 3 months intervention period, from 3038 and 3156 hospitalized adult patients, 249 (59% of them were male, median age = 69 years) and 96 (51% of them were male, median age = 70 years) received parenteral ARs. Total duration of hospitalization of patients on AR was reduced from 28 to 17 days of hospitalization on 100 patient-days (OR = 1.92; 95% CI 1.83–2.01; p < 0.001) with no statistical significant difference in rehospitalisation due to infection of patients that were treated with ARs within 2 months after discharge. Despite short period of time and limited human resources, A-team restrictive interventions rationalised parenteral AR use and led to positive impact on clinical outcomes. These results could help our and other A-teams in similar situation in continuing with the programme to bring more evidence.

## Introduction

With estimated 10 million deaths until 2050, antimicrobial resistance (AMR) will reach the first place of main cause of deaths, before deaths from carcinoma or diabetes [1]. In addition to slow development of new antimicrobial drugs, these led to creation of list of priority pathogens by World Health Organization (WHO) to support research of new and effective drugs [2]. Moreover, to preserve existing antibiotics, WHO also updated their Essential Medicines List for antibiotics, and designated all drugs into access (first line choice), watch and reserve (last line antibiotics). Antibiotic reserves (ARs) should be given as the last line of treatment when other antibiotics no longer work [2]. ARs are useful for a wide range of infections but, because of need to reduce the risk of development of resistance and its relatively high cost, it would be inappropriate to recommend their unrestricted use [3]. Despite this, their use and resistance are increasing worldwide [4-7]. As a result, importance of antimicrobial stewardship teams (A-team) has never been more substantial [8-10].

A cost–benefit analysis showed that maintaining A-team leads to improvements in quality and cost of care with positive clinical outcomes (i.e. by assuring appropriate treatment), compared with standard of care [11]. Additionally, effect of AMR policies seems to be variable. Absence of progress is partly due to insufficient evidence base to inform policymakers about effectiveness, generalizability, and cost-effectiveness of initiatives [12]. Despite evidence of benefits of A-team implementation, it has still not been recognized and applied in Croatia. Some reasons: not enough infectious disease (ID) specialists or incorporated clinical microbiologists in majority of hospitals or no clinical pharmacists included in clinical medicine.

In this study, we aimed to determine whether implementation and multidisciplinary approach of A-team, in a short period of time, compared with period without A-team, would rationalize number of patients on parenteral AR, without negatively affecting clinical outcomes in acute secondary care public hospital with before mentioned obstacles. Achieving the goal could make A-team to continue with the programme and serve as example for other hospitals and countries with limited human resources.

## Main text

### Methods

#### Study design and setting

This was a single-centred cohort before and after study. The study was performed in 325 adult-bed acute secondary care general hospital “Dr. Tomislav Bardek” Koprivnica, Croatia, across all adult wards which covers area with 115 584 inhabitants [13]. Included were all hospitalized patients receiving parenteral ARs which were actively used in hospital and were on basic medical reimbursement list of Croatian health insurance fund (piperacillin/tazobactam, ceftazidime, ceftriaxone, cefepime, meropenem, ertapenem, amikacin, vancomycin and colistin (polymyxin E) as well as ciprofloxacin due to evidence of growing resistance [14, 15]. Chosen were two periods of 3 months (preintervention period from 01. May 2017 to 31. July 2017 and intervention period from 01. September 2017 to 30. November 2017). The A-team intervention started on 01. September 2017.

A-team consisted of multidisciplinary specialists such as 1 FTE ID specialist, 0.5 FTE clinical microbiologist and 0.5 FTE clinical pharmacist who cooperated on daily basis except after working hours, on weekends and holidays. One FTE is defined as full time equivalent of eight working hours from Monday to Friday. Outside that time, an attending physician (a specialist) could prescribe AR, which was reviewed on the first working day by A-team. In that way, there was no delay in the start of antibiotic treatment.

Criteria proposed by Centre for Disease Control and Prevention for appropriate use of antibiotics in hospital settings were described in Standard operative procedure and followed [16, 17].

#### Type and target of the intervention

We chose restrictive intervention due to evidence of more significant immediate effect on prescribing than education and persuasion interventions [18]. It included: a review of medical record by A-team, an interpretation of laboratory susceptibilities from clinical microbiologist, prior audit of ID specialist (bedside consultation) with validation and authorization of prescriptions by clinical pharmacist. The criteria for prescribing AR were: patients with symptoms that were significant or severe (with median definition of fever ≥ 37.5 °C [19]), high risk of complications or the infection is not resolving. This recommendation was communicated to treating physicians, but final commitment of implementing clinical decisions was the responsibility of the treating team. A recommendation was also documented in the medical record.

#### Inclusion and exclusion criteria

All adult inpatients who received parenteral AR were included in the study. Exclusion criteria were patients already under the care of ID physician, being treated in preintervention period or paediatric patients.

#### Assessment of the outcomes

Primary outcomes were number, proportion of patients with ARs prescribed and total duration of hospitalization of patients on ARs per 100 patient days. The number of rehospitalized patients due to infection which were previously treated with AR in 1st, 2nd and 3rd month after discharge was also investigated. Diagnoses for the infection were following according to WHO International Statistical Classification of Diseases and Related Health Problems ICD-10 descriptive codes (ICD) [20]: diseases of respiratory system (J00-J99) and certain infectious and parasitic diseases (A00-B99). Empirical usage and therapeutic medication were not distinguished in this study. Data were collected monthly from hospital computer system.

Secondary outcomes were the duration of treatment with AR prescribed per 100 patient days. Demographic information (age, gender, main ICD at hospital admission, presence of fever, comorbidities and admitted ward) was obtained to assess risk factors for starting AR.

#### Preintervention period

Preintervention period consisted of prescribing ARs on AR computer ordered form in all adult wards. Physician authorized for prescribing AR filled AR form with basic information on patient and drug (patient′s name and date of birth, working diagnosis, name of AR and daily dosage). This authorization was sent in printed form to the hospital pharmacy. The pharmacist and an experienced pharmacy technician checked the validity and dispensed AR for 2 or 3 days of therapy. Every further prolongation of AR therapy required a new authorization by authorized physician and validation from a pharmacist. The pharmacist did not have access to medical record for further information (e.g., allergy)*.*

Preintervention period targeted at the same ARs with the same criteria for prescribing and same primary and secondary outcomes but without A-team.

#### Statistical analysis

Patient characteristics in both periods were compared using Wilcoxon rank-sum test for continuous variables and Fisher exact test for binary categorical variables.

The number of rehospitalization due to infection after 1, 2 and 3 months of discharge were compared using Fisher exact test. Duration of hospitalization of patients on AR and average duration under AR treatment were compared using a Chi-squared test.

All p values only less than 0.05 were considered statistically significant. Statistical analysis was performed in R, version 3.4.4. [21].

### Results

#### Patient characteristics

Baseline characteristics are shown in Table 1. There was no statistically significant difference between baseline characteristics of proportion of patients on ARs in both periods. Median ages of patients on AR were 69 and 70 years in preintervention and intervention period. Most patients on AR had fever and were admitted to Intensive care unit (ICU) with no statistical difference in comorbidities and main ICD diagnosis on admission in both periods.

**Table 1:** Characteristics of patients to whom were prescribed antibiotics reserves (ARs) in pre- and intervention period

Variable	Preintervention period^a^	Intervention period^a^	p
N on AR/overall N	249/3038 (8%)	96/3156 (3%)	< 0.001
Bed-days on AR/overall bed-days	3701/20,902 (18%)	1683/20,722 (8%)	< 0.001
Age, median (min–max)	69 (19–93)	70 (20–89)	0.240
Male sex	146 (59%)	49 (51%)	0.851
Admitted ward			
Internal	190 (76%)	68 (71%)	0.530
Intensive care unit (ICU)	32 (13%)	13 (14%)	0.106
Surgery	18 (7%)	13 (14%)	0.013
Neurology	7 (3%)	2 (2%)	1.000
Psychiatry	2 (1%)	0 (0%)	1.000
Fever	173 (72%)	70 (73%)	0.350
Comorbidities	226 (91%)	87 (94%)	0.931
Main ICD at hospital admission			
Diseases of the respiratory system (J00-J99)	61 (24%)	18 (19%)	0.332
Certain infectious and parasitic diseases (A00–B99)	38 (15%)	15 (16%)	1.000
Diseases of the digestive system (K00–K93)	34 (14%)	12 (12%)	0.863
Malignant neoplasms (C00–C97)	25 (10%)	13 (14%)	0.459
Diseases of the genitourinary system (N00–N99)	21 (8%)	12 (12%)	0.316
Diseases of the circulatory system (I00–I99)	27 (11%)	6 (6%)	0.306
Injuries (S00–T14)	14 (6%)	9 (9%)	0.242
Other	29 (12%)	11 (11%)	0.143

*AR* parenteral antibiotic reserves, *ICD* International Statistical Classification of Diseases and Related Health Problems

^a^All values are absolute frequencies with the percentage in parenthesis if not stated otherwise in a row

#### Preintervention and intervention period

In preintervention period, printed forms for all 249 patients were validated and approved. During intervention, A-team evaluated 354 patients of which 96 (27%) consulted on ARs, while in majority (73%) of patients recommended other antibiotic (which was not AR). The acceptance rate was 100% for all evaluated patients.

#### Results on primary outcomes

Results are shown in Table 2. Duration of hospitalization of patients on AR in preintervention and intervention was 28 and 17 days of hospitalization on 100 patient-days (OR = 1.92; 95% CI 1.83–2.01; p < 0.001), respectively.

**Table 2:** Primary outcomes

Variable	Preintervention period^a^	Intervention period^a^	p
N on AR/overall N	249/3038 (8%)	96/3156 (3%)	< 0.001
Duration of hospitalisation of patients on AR on 100 patient days	28	17	< 0.001
Rehospitalization in			
1st month	13/207 (6)	6/75 (8)	0.597
2nd month	10/203 (5)	7/68 (10)	0.146
3rd month	6/200 (3)	5/67 (7)	0.033

*AR* parenteral antibiotic reserves, *ICD* International Statistical Classification of Diseases and Related Health Problems

^a^All values are absolute frequencies with the percentage in parenthesis if not stated otherwise in a row

#### Secondary outcomes

Duration of treatment with AR prescribed in preintervention and intervention periods were 11 and 4 days per 100 patient days (OR = 2.7; 95% CI 2.5–2.9; p < 0.001), respectively. There was no statistical difference in both periods for risk factors for starting AR which were: male patients, median age of 70 years, internal ward admission, fever, existing comorbidities, disease of the respiratory system or certain infectious and parasitic diseases.

### Discussion

Despite limited human resources of A-team and a short period, this study showed a significant impact on clinical outcomes, focusing on parenteral ARs. Restrictive interventions helped to save last line antibiotics for their future use when they are really needed. The first A-team in Croatia, which consisted of ID specialist, clinical microbiologist and clinical pharmacist, demonstrated an immediate impact of their interventions. Example is reduction of duration of hospitalization of patients in intervention period by nearly 40% (from 28 to 17 days of hospitalization on 100 patient-days) with unaffected rehospitalizations. This brought highly needed evidence of positive effects of interventions on patient-centred outcomes [10, 22]. There was no statistical significance in rehospitalisation of patients on AR due to infection in first 2 months after discharge. However, in the 3rd month after discharge rehospitalisation was raised from 3 to 7% in intervention group which was statistically significant (p = 0.033). One of the reasons could be high risk of mortality due to comorbidity that made patients more vulnerable to influenza in influenza period that had the highest peak during January and February 2018 [23]. Winter was monitoring period for intervention group while preintervention group was followed in autumn period, therefore patients had better chance to stay out of hospital. Second argument could be piperacillin 4 g/tazobactam 0.5 g shortage due to production reasons in intervention period, but that needs further investigation [24].

Complete acceptance rate reflected agreement between A-team, treating physicians and their teams. In addition, this was also in accordance with final step of A-team intervention, which consisted of dispensing AR from hospital pharmacy only after validation from clinical pharmacist with access to medical record. Improving antimicrobial prescribing by clinical pharmacist is in correlation with previous studies and guidelines [25-27]. Multidisciplinary approach has proved to be beneficial in this study for understanding the A-team interventions and therefore accomplishing primary outcomes. Other studies have also proven importance of multidisciplinary approach but in other areas such as preoperative prophylaxis, broad-spectrum antibiotic consumption in various settings or timeliness of antimicrobial therapy in patients with positive blood cultures [28-31].

Risk factors for starting AR can be used in hospitals with resource limitation as a measure of estimation of patients that need special attention.

Furthermore, our aim correlates with WHO Global Action Plan on Antimicrobial Resistance to contain antibiotic resistance, optimize antibiotic treatment and to preserve 'last-resort' antibiotics [32].

This study suggests that A-team restrictive interventions focused on parenteral ARs are useful strategy in improving antimicrobial prescribing without compromising clinical outcomes. Moreover, we proved possibility of achieving our goals despite limited human resources and short period. In addition, results could serve as evidence for including clinical pharmacists in clinical medicine in Croatia. Our research demonstrated the best practice that could be transferred to other national and transnational institutions.

## Limitations

Study was a single centre non-randomized study, which was the first limitation. However, it was difficult to blind investigators or participants in trial of antimicrobial stewardship. Second limitation was two periods of 3 months which were short to prove sustainability, but we demonstrated A-team programme's value by focusing on outcome measures. Third, A-team just focused on AR and not on other antibiotics, which should be investigated in future studies. Nonetheless, we wanted to investigate the effectiveness of A-team on a small but very important group of antibiotics that are costly and under restricted use. In addition, the aim was to show our positive results to managers who could give us more resources to broaden our interventions to other classes of antimicrobials. Finally, we did not investigate AMR due to too short period of follow up, but this is our plan for future.

The future research is needed with a larger multi-centred study, more extended period of intervention, follow up and feedback of A-team with particular emphasis on effectiveness of clinical pharmacy services.

## Data Availability

The data that support the findings of this study are available from general hospital “Dr. Tomislav Bardek” Koprivnica, Croatia but restrictions apply to the availability of these data, which were used under license for the current study, and so are not publicly available. Data are however available from the authors upon reasonable request and with permission of general hospital “Dr. Tomislav Bardek” Koprivnica, Croatia.

## Abbreviations

A-team: antimicrobial stewardship team
AMR: antimicrobial resistance
AR: antibiotic reserve
FTE: full time equivalent
ICD: International Statistical Classification of Diseases and Related Health Problems
ICU: intensive care unit
ID: infectious disease
WHO: World Health Organization
